# A comparative study between a transscleral sustained-release device and eyedrops on intraocular distribution of carteolol hydrochloride

**DOI:** 10.1016/j.heliyon.2023.e14392

**Published:** 2023-03-09

**Authors:** Yoshiko Hashikawa, Yuki Kato, Hirokazu Kaji, Toshiaki Abe, Nobuhiro Nagai

**Affiliations:** a Division of Clinical Cell Therapy, United Centers for Advanced Research and Translational Medicine (ART), Tohoku University Graduate School of Medicine, 2-1 Seiryo-machi, Aoba-ku, Sendai 980-8575, Japan; b Department of Biomechanics, Institute of Biomaterials and Bioengineering, Tokyo Medical and Dental University, 2-3-10 Kanda-Surugadai, Chiyoda, Tokyo 101-0062, Japan

**Keywords:** Controlled release, poly(ethyleneglycol) dimethacrylate, Intraocular drug distribution, Carteolol, Transscleral administration

## Abstract

The objectives of this study were to develop a sustained-release device for carteolol hydrochloride (CH) and investigate any potential difference in the intraocular distribution of this agent between the transscleral administration of the device and treatment with eyedrops. The device was formulated with photocurable resin, poly (ethyleneglycol) dimethacrylate, to fit within the curve of the rabbit eyeball. *In vitro* study showed that CH was released in a sustained-release manner for 2 weeks. The concentration of CH in the retina, choroid/retinal pigment epithelium, sclera, iris, and aqueous humor was determined by high-performance liquid chromatography. Transscleral administration was able to deliver CH to the posterior segment (i.e., retina and choroid/retinal pigment epithelium) rather than the anterior segment (i.e., aqueous humor), while eyedrops delivered CH only to the anterior segment. Transscleral administration could deliver CH to aqueous humor at half the concentration versus treatment with eyedrops and reduced intraocular pressure (IOP) at 1 day after implantation; however, the IOP-lowering effect was not sustained thereafter. In conclusion, transscleral drug delivery may be a useful method for the reduction of IOP. Notably, the aqueous concentration must be equal to that delivered by the eyedrops, and this approach might be preferable for drug delivery to the posterior segment of the eye.

## 1 Introduction

Currently, the most commonly utilized routes for the administration of medications in the treatment of eye diseases are eyedrops and intravitreal injections. The choice of route depends on convenience and compliance, as well as the pharmacokinetic and pharmacodynamic profiles of drugs. We previously developed a transscleral drug delivery device composed of photopolymerized poly (ethyleneglycol) dimethacrylate (PEGDM) [[1], [2], [3]]. The device is designed to be placed on the sclera and fit within the curve of the eyeball, aiming to deliver a drug from the sclera to the retina. The transscleral administration route is a promising minimally invasive approach, offering local sustained delivery to the posterior segment of the eye. We previously reported the safety and efficacy of transscleral administration using a transgenic retinal degeneration model for retinitis pigmentosa [[4], [5], [6]] and a laser-induced choroidal neovascularization model for age-related macular degeneration [7,8]. In addition, an important feature of the device is its ability for long-term release (>1 year) [4]. We seek to extend the range of applications of transscleral administration. It is hypothesized that this transscleral sustained-release device can be utilized for the reduction of intraocular pressure (IOP) in the treatment of glaucoma.

Glaucoma is a leading cause of irreversible blindness worldwide. The reduction of IOP is the only factor and approved therapy for delaying disease progression. Topical antiglaucoma medications have been used for many years. However, the effectiveness of the currently available treatment with eyedrops is limited by poor patient adherence, low bioavailability due to tear fluid turnover, and the occurrence of local and systemic side effects. Thus, various approaches using drug delivery or controlled-release systems have been investigated to overcome these limitations. Yan et al. assessed a bimatoprost imprinted silicone contact lens which was able to release the drug for up to 60 h [9]. Samy et al. reported an intraocular polycaprolactone thin-film that released timolol and brimonidine, providing an IOP-lowering effect for 3 months *in vivo* [10]. Nguyen et al. investigated an intracameral biodegradable thermogel releasing pilocarpine and reported its anti-inflammatory effect for >80 days [11].

The objectives of this study were to design a sustained-release device for carteolol hydrochloride (CH) and evaluate its *in vitro* release profile, to compare the intraocular distribution of CH between the transscleral administration of the device and conventional eyedrop administration, and to assess the potential of transscleral administration to reduce intraocular pressure (IOP).

## 2 Materials and methods

### 2.1 Preparation of the device containing carteolol hydrochloride (CH)

The device for the sustained release of CH was formulated with PEGDM to fit within the curve of the rabbit eyeball. PEGDM with Mn 736 (PEGDM-14G; Shin-Nakamura Chemical, Japan) and PEGDM with Mw 330 (PEGDM-4G; Shin-Nakamura Chemical, Wakayama materials, Japan) containing 1% 2-hydroxy-2-methylpropiophenone (Tokyo Chemical Industry, Tokyo, Japan) as a photo-initiator were used as starting. CH (Tokyo Chemical Industry) was dissolved in a mixture of 40% PEGDM-14G/60% PEGDM-4G (abbreviated as P40) at a concentration of 500 mg/mL. The mixture was poured into a microfabricated polydimethylsiloxane (Silpot 184 W/C; Dow Corning Toray, Tokyo, Japan) mold. This was followed by photopolymerization with ultraviolet (365 nm) light for 240 s at an intensity of 38 mW/cm^2^ (LC8; Hamamatsu Photonics, Shizuoka, Japan). The dimensions of the device were as follows: 12 mm length × 4.4 mm width × 0.7 mm height. The volume of the drug formulation was 48 μL, and the loading amount was 2.4 mg.

### 2.2 *In vitro* release study

The devices were incubated in 2 mL of phosphate-buffered saline (PBS) at 37 °C. The amount of CH that diffused out of the device was measured using high-performance liquid chromatography (HPLC) (Prominence; Shimadzu, Tokyo Japan). A YMC-pack ODS-AQ column (YMC, Tokyo, Japan) was used as a reversed-phase analytical column. The mobile phase was methanol/water containing 1% trifluoroacetic acid (30:70, vol/vol), and was delivered isocratically at 1 mL/min. The chromatograms were monitored at 220 nm wavelength. The PBS was replenished during the course of the release study to ensure that the concentration of CH was maintained <20% of its saturation value at all times. The results were determined using a standard curve.

### 2.3 Animals

Male Japanese white rabbits (Kumagai Shigeyasu Co., Sendai, Japan) (weight: 1.8 kg) were used in this study. All animals were maintained and cared for in accordance with the Association for Research in Vision and Ophthalmology Statement for the Use of Animals in Ophthalmic and Vision Research. The study was approved by the Institutional Animal Care and Use Committee of the Tohoku University Environmental & Safety Committee (approved number: 2019MdA-306).

### 2.4 Implantation and eyedrops

The rabbits were anesthetized with ketamine hydrochloride (90 mg/kg) and xylazine hydrochloride (10 mg/kg). Their ocular surfaces were anesthetized with a topical instillation of 0.4% oxybuprocaine hydrochloride. A 5 × 5 mm paralimbal conjunctival incision was performed at the upper temporal limbus. The device was inserted between the conjunctiva and sclera, and the front head of the device was placed immediately beside the optic nerve head. The device positions were confirmed after enucleation at the end of the experiments. The peripheral region was sutured onto the sclera with 7–0 suture silk (Alcon, Tokyo, Japan) to tightly fix the devices onto the sclera. The incision was closed with 9–0 suture silk (Alcon, Tokyo, Japan), and antibiotic ophthalmic ointment (Santen Pharmaceutical, Osaka, Japan) was inserted into the conjunctiva. For the eyedrops, CH ophthalmic solution 2% (Wakamoto Pharmaceutical, Tokyo, Japan) was applied at a volume of 50 μL.

### 2.5 Intraocular distribution of CH

The concentration of CH in the retina, choroid/retinal pigment epithelium (RPE), sclera, iris, and aqueous humor was determined by HPLC. After the aqueous humor was collected, the eyes were enucleated. Next, the retina, choroid/RPE, sclera, and iris were separated. Except for the aqueous humor, the tissues were homogenized in PBS. Following centrifugation at 14,000×*g* for 2 min at room temperature, the supernatants were collected. The HPLC conditions were identical to those set for the *in vitro* release study. The results were obtained using a standard curve. To determine the intraocular distribution of CH, the devices or eyedrops were applied to both eyes. Six eyes were collected at 1 and 2 days after transscleral administration or at 1 h after treatment with eyedrops.

### 2.6 IOP measurement

The IOP was measured before and at 1 and 3 days after implantation using a tonometer (Tonovet M.E. Technica, Tokyo, Japan). In the case of treatment with eyedrops, the IOP was measured before and at 15, 30, 60, 90, and 120 min after instillation. For IOP measurement, the devices or eyedrops were applied to the left eyes. The right eyes were treated with placebo devices or PBS eyedrops and served as control.

### 2.7 Statistical analysis

Statistical analysis was conducted using the BellCurve for Excel (Social Survey Research Information, Tokyo, Japan) software, and the results were presented as the mean ± standard deviation. The Student's *t*-test was used for statistical analysis. *P*-values <0.05 denoted statistically significant differences.

## 3 Results

### 3.1 Device fabrication and *in vitro* release study

**Fig. 1:**
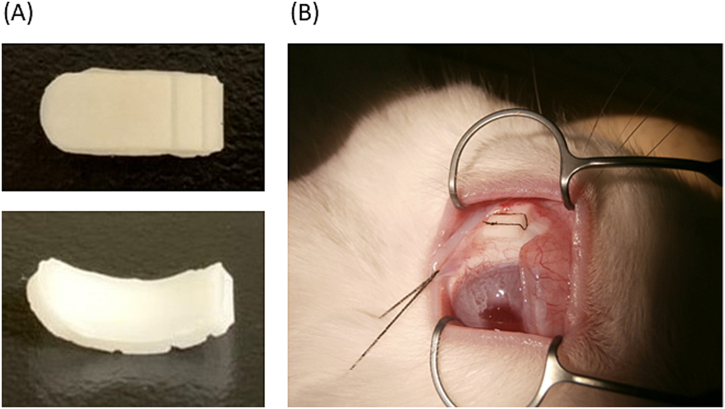
(A) Sustained CH-releasing device. (B) Device placed and sutured on the sclera of the rabbit. CH, carteolol hydrochloride.

**Fig. 2:**
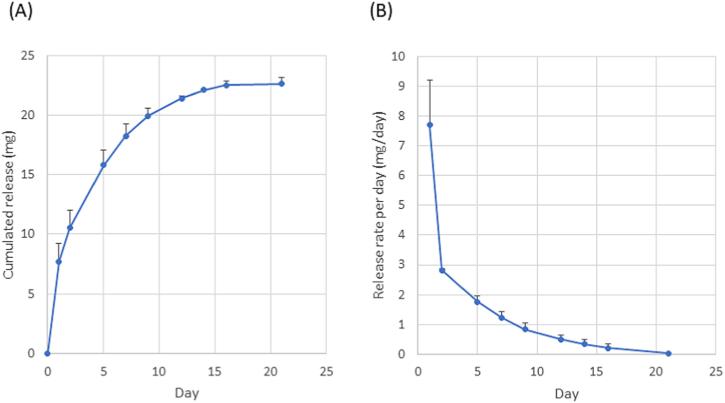
*In vitro* release profile of carteolol hydrochloride (CH) from the DDS. (A) Cumulated release and (B) release rate per day. Data points denote the mean ± SD (n = 5). SD, standard deviation.

The CH-loaded device is shown in Fig. 1A. It includes grooves for sutures, which assist in fixing the device on the sclera. The curvature radius was 6 mm; this was similar to the axial length of the rabbit eyeball. Fig. 1B shows a device sutured on the sclera. After implantation, there was no movement of the device from the implantation site. The *in vitro* release profiles are shown in Fig. 2. A burst release was observed on the first day, which was followed by sustained release (Fig. 2A). The release rate gradually decreased and reached the plateau at the loading amount of ∼2.4 mg (Fig. 2B).

### 3.2 Intraocular distribution of CH

**Fig. 3:**
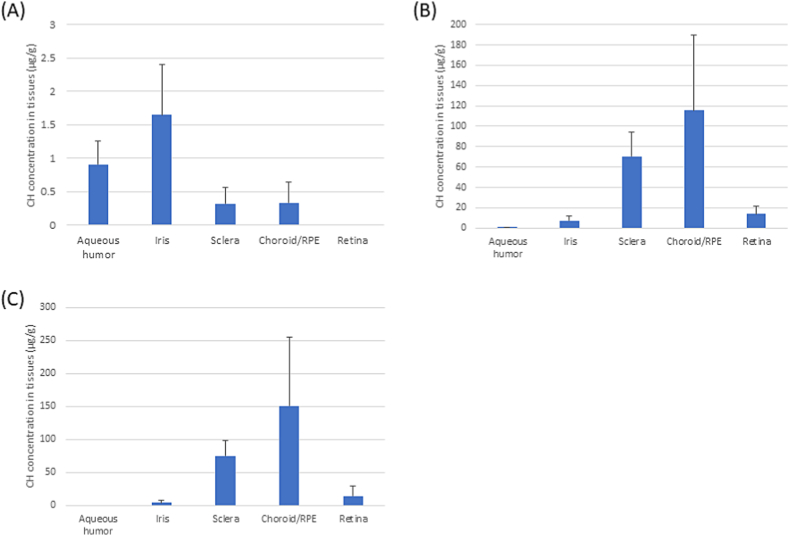
Intraocular amount of CH in tissues after treatment with (A) eyedrops for 1 h, and transscleral administration for (B) 1 day and (C) 2 days. Data points denote the mean ± SD (n = 6). CH, carteolol hydrochloride; RPE, retinal pigment epithelium; SD, standard deviation.

**Fig. 4:**
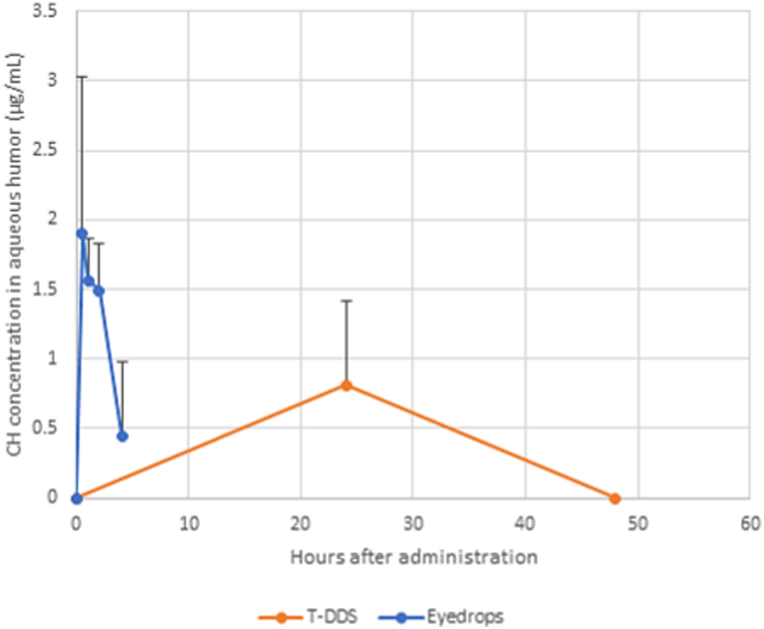
Time course of CH concentration in the aqueous humor after treatment with eyedrops and transscleral administration (T-DDS). Data points denote the mean ± SD (n = 6). CH, carteolol hydrochloride; SD, standard deviation; T-DDS, transscleral drug delivery system.

The distribution of CH in the intraocular tissues is shown in Fig. 3. In the case of treatment with eyedrops, CH was mainly distributed to the anterior segment (i.e., aqueous humor and iris) (Fig. 3A). However, in the case of transscleral administration, CH was distributed to the posterior segment (i.e., choroid/RPE and retina) (Fig. 3B and C). Fig. 4 shows that the maximal concentration (C_max_) of CH delivered by transscleral administration in the aqueous humor was approximately half of that delivered by eyedrops. The area under the curve values for the two routes of administration were 39.1 and 4.8 μg/mL・h, respectively.

We sought to investigate the drug availability associated with each method. For this purpose, we calculated the transport rate to tissues of treatment with eyedrops at 1 h and transscleral administration at 1 and 2 days. The amount of CH in the tissues was divided by the released amount at the indicated timepoint from Fig. 1 (1 mg/eyedrops at 1 h, 7.7 mg/transscleral administration at 1 day, and 10.5 mg/transscleral administration at 2 days). In the case of treatment with eyedrops, the transport rates to the aqueous humor and iris were higher than those noted in other tissues, and these rates were ∼0.02% (Table 1). Transscleral administration showed a lower transport rate to aqueous humor (0.001%) (Table 2). However, the transport rate to the retina was higher than that observed after treatment with eyedrops, and the rates were 0.008% and 0.007% at 1 day (Table 2) and 2 days (Table 3), respectively. Treatment with eyedrops did not transport CH to the retina (Table 1). Moreover, the transport rate to the choroid/RPE was also higher after transscleral administration versus treatment with eyedrops. The rates for transscleral administration were 0.049% and 0.048% at 1 day (Table 2) and 2 days (Table 3), respectively, while that of treatment with eyedrops was 0.0012% (Table 1).

**Table 1:** Transport rate from eyedrops to tissue at 1 h.

Tissue	Total CH amount in tissues (μg)	Transport rate from eyedrops (1 mg) to tissue
Aqueous humor	0.215 ± 0.111	0.0215%
Iris	0.227 ± 0.154	0.0227%
Sclera	0.088 ± 0.059	0.0088%
Choroid/RPE	0.012 ± 0.011	0.0012%
Retina	0 ± 0	0.0000%

Values are the mean ± SD; n = 4. CH, carteolol hydrochloride; RPE, retinal pigment epithelium.

**Table 2:** Transport rate from transscleral administration to tissues at 1 day.

Tissue	Total CH amount in tissues (μg)	Transport rate from DDS (7.7 mg) to tissue
Aqueous humor	0.081 ± 0.067	0.0011%
Iris	1.022 ± 0.498	0.0133%
Sclera	17.11 ± 3.376	0.2222%
Choroid/RPE	3.818 ± 2.921	0.0496%
Retina	0.643 ± 0.058	0.0083%

Values are the mean ± SD; n = 4. CH, carteolol hydrochloride; DDS, drug delivery system; RPE, retinal pigment epithelium.

**Table 3:** Transport rate from transscleral administration to tissues at 2 days.

Tissue	Total CH amount in tissues (μg)	Transport rate from DDS (10.5 mg) to tissue
Aqueous humor	0 ± 0	0.0000%
Iris	0.560 ± 0.484	0.0053%
Sclera	19.88 ± 7.003	0.1894%
Choroid/RPE	5.047 ± 3.131	0.0481%
Retina	0.780 ± 0.910	0.0074%

Values are the mean ± SD; n = 4. CH, carteolol hydrochloride; DDS, drug delivery system; RPE, retinal pigment epithelium.

### 3.3 IOP measurement

**Fig. 5:**
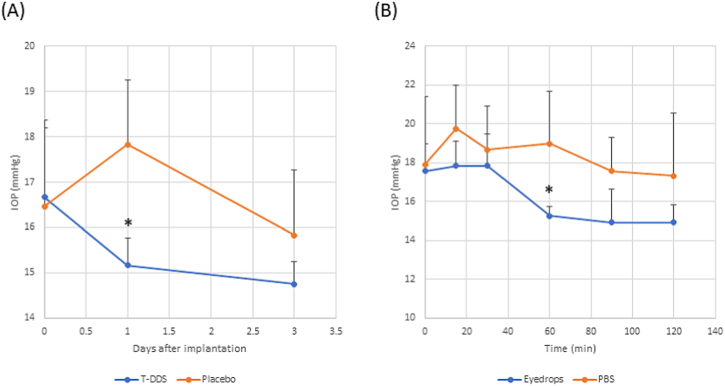
IOP in the eyes treated with (A) transscleral administration (T-DDS) and (B) eyedrops. Placebo device or PBS eyedrop was used as control. Data points denote the mean ± SD (n = 4). IOP, intraocular pressure; PBS, phosphate-buffered saline; SD, standard deviation; T-DDS, transscleral drug delivery system.

Fig. 5 shows the IOP of rabbits after transscleral administration and treatment with eyedrops. Significant decrease in IOP compared with the control was observed for both transscleral administration (Fig. 5A) and treatment with eyedrops (Fig. 5B) at 1 day and 1 h, respectively. After these time points, there was no statistically significant difference observed between the treatments and control.

## 4 Discussion

There are several types of controlled-release systems for the delivery of antiglaucoma drugs, including contact lens [9], thin-film [10], and thermogels [11]. However, there are few reports on the effectiveness of CH-loaded drug delivery systems [12,13]. All existing systems are aimed at delivering drugs to the anterior segment; thus, previously, there were no transscleral route-oriented systems. We found that transscleral administration could reduce the IOP at 1 day, though the IOP-lowering effect was not sustained thereafter. In contrast, the drug release was sustained for 2 weeks. This may be related to the reduced CH concentration in the aqueous humor 2 days after implantation of the device (Fig. 4). The mechanism underlying the ocular hypotensive action of CH is most likely a decrease in aqueous humor by blocking beta adrenoceptors in the ciliary body in the eye [14]. Thus, inadequate transport to the anterior segment by transscleral route would reduce the IOP-lowering effect. The transport rate to the aqueous humor of treatment with eyedrops was 20-fold higher than that of transscleral administration (Table 1 vs. Table 2). Considering that the device was able to reduce the IOP at 1 day, the release of >7.7 mg per day (observed at 1 day) via the transscleral route should be maintained in our experimental condition.

In contrast, transscleral administration offered the advantage of drug delivery to the posterior segment. We found that, unlike treatment with eyedrops, the transscleral sustained release could deliver CH to the retina. The concentration of CH in the choroid/RPE delivered by transscleral administration was 40-fold higher than that delivered by treatment with eyedrops (Table 1 vs. Table 2). It is well established that drug access by eyedrops is generally limited due to static (i.e., cornea, sclera, and RPE) and dynamic (i.e., tear dilution, conjunctival and choroidal blood flow, and lymphatic clearance) barriers as well as efflux pumps [15]. Thus, less drug reached the posterior segment of the eye after treatment with eyedrops. For the purpose of drug delivery to the posterior segment, transscleral administration is a preferable route owing to the local availability to the retina and minimal invasiveness to the intraocular tissues. Despite the availability of numerous types of systems for the delivery of drugs to the posterior segment, these systems require placement of an implant in the vitreous body. Hence, surgery is necessary for the placement or replacement of the implant, which could damage intraocular tissues. Our episcleral implant is easily placed or replaced on the sclera through minor surgery [5,6]. Thus, transscleral administration might be suitable for sustained drug delivery to the posterior segment.

The substrate of the device is photopolymerized PEGDM, which is non-biodegradable in the body. Previous evidence regarding long-term implantation on the sclera has shown that this material is bio-inert, biocompatible, and mechanically stable for up to 19 months in rabbits [16]. Currently, we have initiated a clinical study of an unoprostone-releasing device for patients with retinitis pigmentosa. A major limitation of the PEGDM-based implant is the need for replacement following drug depletion. Concerning replacement, biodegradable implants (e.g., intracameral biomatoprost implant) may be tolerated [17]. Also, intraocular implants are associated with a risk of complications in the eye. Therefore, several challenges must be overcome prior to establishing the use of sustained-release systems for the treatment of glaucoma. Particularly, differences between the delivery routes in terms of drug efficacy and safety should be evaluated, and these profiles should be compared with that of conventional topical therapy. Improvement of patient adherence by the use of these devices may drastically impact disease progression and patient quality of life.

Given the limited efficacy in reducing IOP and the advantage of drug delivery to the posterior eye segment through the device, transscleral administration of neuroprotective agents may offer a means of protecting the optic nerve. Various strategies are being explored to prevent the degeneration of retinal ganglion cells by neuroprotective agents [18]. In the future, we plan to assess the effectiveness of a neuroprotective agent-release device in a nerve crush model [19]. Further study of sustained-release systems holds the potential to transform the treatment of glaucoma.

## 5 Conclusion

Transscleral administration that can sustain drug release at >7 mg/day is able to reduce IOP. Considering the drug availability in the posterior segment of the eye, transscleral administration appears to be preferable versus treatment with eyedrops. Sustained release could improve patient adherence and drug efficacy for diseases in the posterior segment of the eye.

## Author contribution statement

Nobuhiro Nagai: Conceived and designed the experiments; Performed the experiments; Analyzed and interpreted the data; Contributed reagents, materials, analysis tools or data; Wrote the paper.

Yoshiko Hashikawa: Conceived and designed the experiments; Performed the experiments; Analyzed and interpreted the data; Wrote the paper.

Yuki Kato: Performed the experiments; Analyzed and interpreted the data.

Hirokazu Kaji: Analyzed and interpreted the data.

Toshiaki Abe: Analyzed and interpreted the data; Contributed reagents, materials, analysis tools or data.

## Funding statement

Dr Nobuhiro Nagai was supported by 10.13039/501100002241Japan Science and Technology Agency [JPMJST1917].

## Data availability statement

Data will be made available on request.

## Declaration of interest's statement

The authors declare that they have no known competing financial interests or personal relationships that could have appeared to influence the work reported in this paper.

## Competing financial interests

The authors have no conflict of interest to declare.
